# The Portuguese Albuminuria Study: national insights into prevalence and risk factors

**DOI:** 10.1093/ckj/sfaf240

**Published:** 2025-07-30

**Authors:** Ana Carina Ferreira, Ana Farinha, Edgar Almeida

**Affiliations:** Nephrology Department, Hospital de Curry Cabral, ULS São José, Lisbon, Portugal; Nova Medical School, Nova University of Lisbon, Lisbon, Portugal; Nephrology Department, ULS Arrábida, Setúbal, Portugal; Faculdade de Medicina da Universidade do Porto, Oporto, Portugal; Nephrology Department, Hospital Beatriz Ângelo, Loures, Portugal; Faculdade de Medicina, Universidade de Lisboa, Lisbon, Portugal

**Keywords:** albuminuria, chronic kidney disease, screening

## Abstract

**Background:**

Chronic kidney disease (CKD) is a global health concern associated with increased cardiovascular risks. In Portugal, the high burden of CKD highlights the urgent need for early detection strategies. The primary aim of this study was to assess the presence of albuminuria in the Portuguese general population while raising awareness of CKD.

**Methods:**

An epidemiological, cross-sectional study screened 601 individuals for albuminuria using urine test strips, employing a door-to-door approach across the five regions of mainland Portugal (screening study), with 592 valid results included in the final analysis. In parallel, an awareness campaign distributed 17 000 urine test strips, with 704 participants submitting their results through an online platform (awareness study).

**Results:**

The screening occurred in a healthy population, with >70% (screening study) and >87% (awareness study) of the individuals reporting no known personal health history. The presence of albuminuria was detected in 5.1% of the screening study population and 3.4% of the awareness study participants. In both studies, significant associations were found between albuminuria and risk factors, such as age, education level, CKD and non-steroidal anti-inflammatory drug use. Regional disparities were also observed. In the screening study, multivariate analysis identified education level (*P* = .011), CKD (*P* < .0001) and autoimmune diseases (*P* = .009) as independent predictors of albuminuria.

**Conclusions:**

These findings highlight albuminuria as a critical early marker for CKD and cardiovascular risks. The results support the need for targeted screening and public health initiatives, particularly in high-risk and younger populations.

## GRAPHICAL ABSTRACT

**GRAPHICAL ABSTRACT:**
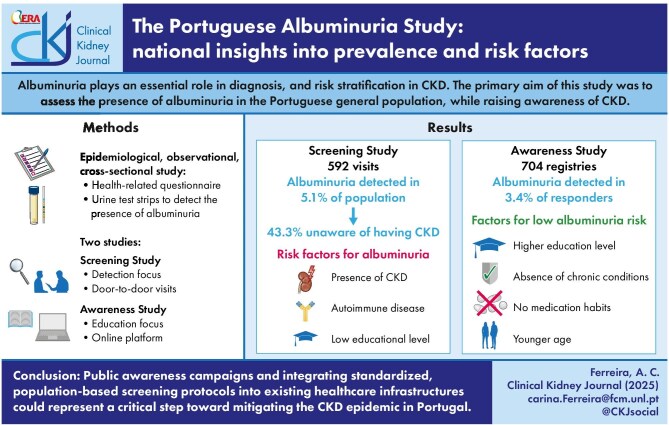

KEY LEARNING POINTS **What was known:**

- Chronic kidney disease (CKD) is a global health problem, affecting 1 in 10 adults. Early detection is key to prevent CKD progression and related cardiovascular events.
- In Portugal, albuminuria screening is recommended for high-risk populations, such as individuals with hypertension and diabetes, potentially leaving undiagnosed cases in the general population.
- No prior national study has evaluated albuminuria prevalence and incidence in the Portuguese general population.

**This study adds:**

- This was the first national study aiming to estimate the prevalence of albuminuria in the Portuguese general population, highlighting that 5% of individuals may have early kidney disease, including younger, lower-risk populations.
- The study identifies regional disparities in albuminuria prevalence, with the North most affected, supporting the need for regional tailored public health interventions.
- The awareness campaign performed in this study demonstrates the feasibility of combining public engagement with health data collection, offering a model for increasing CKD awareness and identifying undiagnosed cases.

**Potential impact:**

- The findings reinforce albuminuria as a key marker of CKD and support the integration of routine screening, especially for high-risk individuals in primary care.
- The study provides robust national data to support national policies promoting early CKD detection and prevention.

## INTRODUCTION

Chronic kidney disease (CKD) is a silent epidemic affecting millions globally, with early stages often undetected. It impacts 1 in 10 adults, and CKD is projected to become the fifth leading cause of years of life lost worldwide by 2040 [1]. Beyond its direct impact, CKD significantly increases the risk of cardiovascular disease (CVD), the primary cause of death in developed countries.

Portugal has the highest rate of dialysis patients in Europe and ranks eighth globally [2, 3], underscoring the urgent need for effective and proactive interventions [4–6]. Albuminuria, a key early marker of both CKD and CVD, plays an essential role in diagnosis, risk stratification and clinical management of the disease [7].

In Portugal, albuminuria screening is currently recommended in primary care settings for high-risk patients, namely those with hypertension and diabetes. While this approach prioritizes those most likely to progress to advanced CKD or experience cardiovascular events, broader population-level screening may uncover undiagnosed cases, enabling timely and potentially cost-effective interventions [8, 9].

Despite its clinical relevance, no study has yet evaluated albuminuria prevalence across the general population in Portugal. This study fills the gap in existing research, aiming to assess albuminuria prevalence (primary aim) while raising awareness of CKD among the Portuguese population (secondary aim).

## MATERIALS AND METHODS

### Study design

This was an epidemiological, observational, cross-sectional and descriptive study conducted in mainland Portugal (excluding the islands) during the second semester of 2023.

The study comprised two components, a screening study and an awareness study, both employing a health-related questionnaire and urine test strips to detect the presence of albuminuria. The questionnaire included sociodemographic information (e.g. age, sex and education), anthropometric measures [e.g. weight, height and blood pressure (BP)] and medical history (e.g. CKD, diabetes, hypertension, dyslipidaemia, stroke, autoimmune diseases, coronary heart disease, heart failure and chronic infections, as well as medication use). Albuminuria was defined as a urinary albumin concentration ≥0.12 g/l, according to the test strip specifications.

#### Data collection: screening study

Adults >18 years of age who agreed to participate voluntarily were recruited on-site through unannounced door-to-door visits. Recruitment was conducted across the five regions of mainland Portugal (North, Centre, Lisbon and Tagus Valley, Alentejo and Algarve) to ensure balanced national representation. Stratified sampling was applied based on sex and age groups, aligning with regional sociodemographic profiles to reduce selection bias. The islands (Azores and Madeira) were excluded due to logistical limitations that prevented door-to-door recruitment and test strip distribution.

The screening component included completing a structured questionnaire and performing a urine test strip. Trained interviewers interpreted the test strip results to ensure consistency and reliability.

#### Data collection: awareness study

A dedicated website was developed to provide educational content on CKD and albuminuria, including risk factors and symptoms, along with access to the health-related questionnaire and a portal for anonymous self-reporting of urine test strip results.

Information cards and urine test strips were distributed using convenience sampling during screening visits (each participant received multiple urine test strips and awareness cards to share with relatives and friends), as well as at events (e.g. fairs, congresses), pharmacies and workplaces to engage a broad demographic spectrum. It was anticipated that <5% of recipients would upload their data, but the primary goal was awareness raising.

### Statistical analysis

The primary outcome in both studies was the presence of albuminuria. Albuminuria prevalence was calculated as the proportion of individuals with test strip values of 0.12 g/l relative to the total study population. Predictor variables included age, sex, education, weight, height, BP, diabetes, hypertension, autoimmune diseases, chronic infections and medication use.

Both studies employed univariate descriptive analysis. Continuous variables were summarized using measures of central tendency and dispersion. Normality was assessed using Kolmogorov–Smirnov and Shapiro–Wilk tests at a .05 significance level. Categorical variables were analysed using absolute and relative frequencies. In both studies, associations between albuminuria and categorical variables were evaluated using the chi-squared test. For continuous variables the Wilcoxon–Mann–Whitney test or Student's *t*-test was used, according to normality, at a significance level of .05.

A multivariate logistic regression analysis was conducted in the screening study to identify independent predictors of albuminuria. Variable selection was based on statistically significant associations from univariate analysis, including age group, region, education level, CKD, autoimmune disease and medication use.

Specific cases with missing data were retained in the analysis: the screening study included 3 participants with missing information on education level and 7 with missing data on region, while the awareness study included 17 participants with missing information on education level and 3 with missing data on region.

## RESULTS

### Screening study

**Figure 1:**
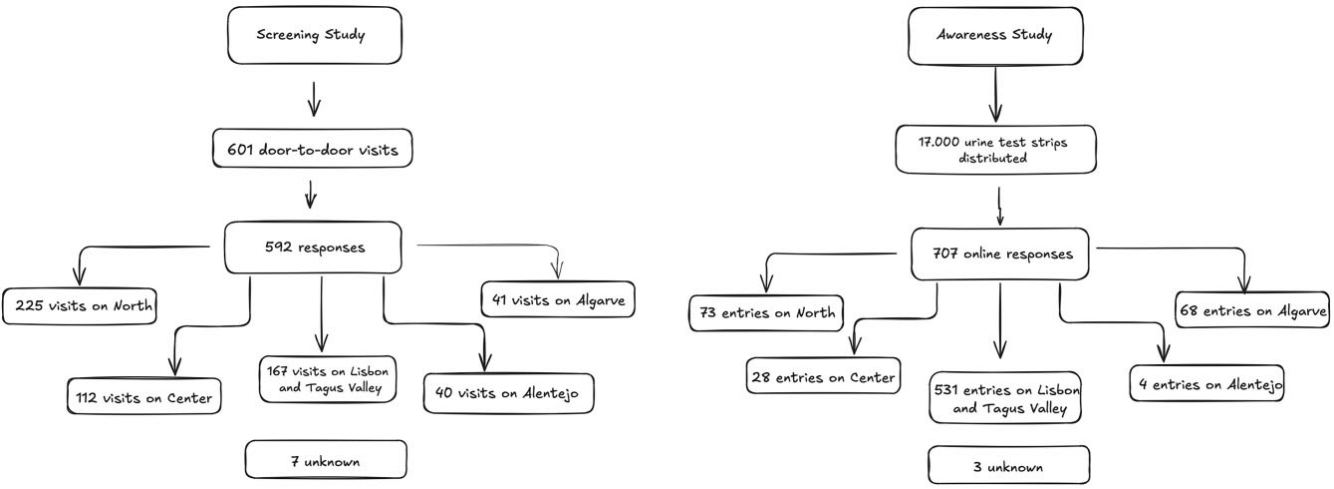
Flow chart of **(a)** the screening study and **(b)** the awareness study.

The flow chart of the screening study is presented in Fig. 1a. Of the 601 visits, 592 were considered complete and included in the final analysis. Table 1 shows the distribution by region, stratified by sex and age group.

**Table 1:** Distribution by region, stratified by sex and age group in the screening study.

		Portugal mainland				
Age group (years)	Sex	North	Centre	LTV	Alentejo	Algarve
<40	Female	42	17	31	7	16
	Male	31	19	28	7	8
40–65	Female	51	23	32	7	4
	Male	36	21	30	7	5
>65	Female	32	16	25	6	4
	Male	33	16	20	6	4

LVT: Lisbon and Tagus Valley.

Missing values for Portugal regions, *n* = 7.

The population included a balanced distribution across younger [<40 years old: *n* = 210 (35.5%)] and middle-aged [40–65 years old: *n* = 218 (36.8%)] groups, with a median age of 48 years [interquartile range (IQR 32–67)]. A slight predominance of females [*n* = 318 (53.7%)] was observed, as well as individuals with secondary education [*n* = 206 (34.8%)]. The median weight was 69 kg (IQR 62–78), the median systolic BP was 120 mmHg (IQR 120–130) and the median diastolic BP was 80 mmHg (IQR 70–80). Table 2 summarizes the sociodemographic and clinical characteristics of the study population.

**Table 2:** Sociodemographic and clinical characteristics of the population in the screening study.

Variable	Category	Screening study, *n* (%)	Awareness study, *n* (%)
Age group (years)	<40	210 (35.5)	388 (54.9)
	40–65	218 (36.8)	207 (29.3)
	>65	164 (27.7)	112 (15.8)
Sex	Female	318 (53.7)	401 (56.7)
	Male	274 (46.3)	304 (43.3)
Education (Screening: missing values, *n* = 3)	Basic education	209 (35.3)	125 (17.5)
	Secondary education	206 (34.8)	268 (37.9)
	≥Bachelor's degree	174 (29.4)	298 (42.1)
Kidney disease	No	563 (95.1)	699 (98.9)
	Yes	29 (4.9)	8 (1.1)
Diabetes	No	549 (92.8)	683 (96.6)
	Yes	43 (7.2)	24 (3.4)
Dyslipidaemia	No	571 (96.5)	603 (99.4)
	Yes	21 (3.5)	4 (0.6)
Stroke	No	583 (98.5)	693 (98.0)
	Yes	9 (1.5)	14 (2.0)
Myocardial ischaemic disease	No	586 (99.0)	704 (99.6)
	Yes	6 (1.0)	3 (0.4)
Congestive cardiac disease	No	575 (97.1)	699 (98.9)
	Yes	17 (2.9)	8 (1.1)
Arterial disease	No	566 (95.6)	706 (99.9)
	Yes	26 (4.4)	1 (0.1)
Autoimmune disease	No	514 (86.8)	692 (97.9)
	Yes	78 (13.2)	15 (2.1)
Infections	No	586 (99.0)	703 (99.4)
	Yes	6 (1.0)	4 (0.6)
No disease	No	176 (29.7)	89 (12.6)
	Yes	416 (70.3)	618 (87.4)
Albuminuria	<0.08	341 (57.6)	457 (64.6)
	0.08	221 (37.3)	226 (32)
	0.12	30 (5.1)	24 (3.4)
Angiotensin-converting enzyme inhibitor	No	568 (96.0)	700 (99.0)
	Yes	24 (4.0)	7 (1.0)
Angiotensin receptor blocker	No	573 (96.8)	702 (99.3)
	Yes	19 (3.2)	5 (0.7)
SGLT2 inhibitor	No	554 (93.6)	692 (97.9)
	Yes	38 (6.4)	15 (2.1)
Calcium channel blocker	No	573 (96.8)	696 (98.4)
	Yes	19 (3.2)	11 (1.6)
NSAID	No	514 (86.8)	672 (95)
	Yes	78 (13.2)	35 (5)
No medication	No	131 (22.1)	64 (9.1)
	Yes	461 (77.9)	643 (90.6)

More than 70% of participants [*n* = 416 (70.3%)] reported no known personal medical history, although autoimmune diseases [*n* = 78 (13.2%)] and diabetes [*n* = 43 (7.2%)] were the most frequently reported conditions among those who did. Regarding medication use, 13.2% reported taking non-steroidal anti-inflammatory drugs [NSAIDs; *n* = 78 (13.2%)], while 6% were on sodium–glucose co-transporter 2 (SGLT2) inhibitors [*n* = 38 (6.4%)].

Albuminuria was detected in 30 individuals, yielding a prevalence of 5.1%. Among these, >40% [*n* = 13 (43.3%)] were unaware of having CKD. The highest regional prevalence was observed in the North (Table 3), and among individuals without a prior CKD diagnosis, albuminuria was more frequently observed in the Algarve and Centre regions (Table 4).

**Table 3:** Prevalence of albuminuria, stratified by region.

Region	Screening study prevalence, % (95% CI)	Awareness study prevalence, % (95% CI)
North	9.33 (5.87–13.91)	5.48 (1.51–13.44)
Centre	4.46 (1.48–10.20)	0
Lisbon and Tagus Valley	1.20 (0.15–4.39)	3.77 (2.32–5.76)
Alentejo	0	0
Algarve	4.88 (0.60–16.53)	0

**Table 4:** Prevalence of albuminuria in persons without known kidney disease, stratified by region.

Region	Screening study prevalence, % (95% CI)	Awareness study prevalence, % (95% CI)
North	2.90 (1.07–6.20)	0
Centre	3.85 (1.06–9.56)	0
Lisbon and Tagus Valley	0.63 (0.02–3.45)	3.42 (2.04–5.34)
Alentejo	0	0
Algarve	4.88 (0.66–16.53)	0

Univariate analysis (Table 5) revealed statistically significant associations between albuminuria and age group (*P* < .001), education level (*P* < .001), presence of CKD (*P* < .001), autoimmune diseases (*P* < .001), chronic infections (*P* < .001) and use of NSAIDs (*P* < .001). Absence of chronic conditions or no medications were both associated with a lower likelihood of albuminuria.

**Table 5:** Bivariate analysis of independent variables versus the presence of albuminuria in the screening study.

		Albuminuria		
Variable	Category	No, *n* (%)	Yes, *n* (%)	*P*-value
Age group (years)	<40	207 (36.8)	3 (10)	<.001
	40–65	212 (37.7)	6 (20)	
	>65	143 (25.4)	21 (70)	
Education	Basic	201 (35.8)	5 (16,7)	<.001
	Secondary	173 (30.8)	1 (3.3)	
	≥Bachelor's degree	2 (0.4)	1 (3.3)	
CKD	No	550 (97.9)	13 (43.3)	<.001
	Yes	12 (2.1)	17 (56.7)	
Autoimmune disease	No	499 (88.8)	15 (50)	<.001
	Yes	63 (11.2)	15 (50)	
Infection	No	559 (99.5)	27 (90)	<.001
	Yes	3 (0.5)	3 (10)	
NSAIDs	No	498 (88.6)	16 (53.3)	<.001
	Yes	64 (11.4)	14 (46.7)	
Disease	Without	413 (73.5)	3 (10)	<.001
	With	149 (26.5)	27 (90)	
Medication	Without	446 (79.4)	15 (50)	.001
	With	116 (20.6)	15 (50)	
Portugal regions	North	204 (36.3)	21 (70)	<.001
	Centre	107 (19)	5 (16.7)	
	Lisbon and Tagus Valley	165 (29.4)	2 (6.7)	
	Alentejo	40 (7.1)	0 (0)	
	Algarve	39 (6.9)	2 (6.7)	

In multivariate logistic regression analysis, education level (*P* = .011), CKD (*P* < .0001) and autoimmune diseases (*P* = .009) remained independently associated with the presence of albuminuria.

### Awareness study

The flow chart of the awareness study is presented in Fig. 1b.

A total of 17 000 urine test strips were distributed, resulting in 704 individuals registering their responses through the online platform. The population was predominantly younger, with 54.9% <40 years of age. The median age was 36 years (IQR 26–55). Women represented 56.7% of the participants and 42.1% had a bachelor's degree or higher. The median weight was 72 kg (IQR 64–80), the median systolic BP was 120 mmHg (IQR 120–130) and the median diastolic BP was 80 mmHg (IQR 70–85). Table 2 presents the sociodemographic and clinical characteristics of the population.

Participants reported a low prevalence of chronic conditions. More than 87% indicated no personal history of illness, with diabetes (3.4%) and autoimmune diseases (2.1%) being the most reported. Regarding medication use, 5% were on NSAIDs and 2.1% reported taking SGLT2 inhibitors.

Albuminuria was detected in 24 individuals, corresponding to a prevalence of 3.4%. Regional prevalence was highest in the North and in the Lisbon and Tagus Valley region. Among individuals unaware of having CKD, cases were primarily observed in Lisbon and Tagus Valley region (Tables 3 and 4).

Univariate analysis revealed significant associations between albuminuria and age group (*P* = .015) and education level (*P* < .001). Health-related factors associated with albuminuria included known CKD (*P* < .001), diabetes (*P* = .04) and cerebrovascular disease (*P* = .01). In addition, the use of certain medications, such as angiotensin receptor blockers (*P* = .01), SGLT2 inhibitors (*P* = .012) and NSAIDs (*P* = .005), showed statistically significant associations.

## DISCUSSION

This study represents the first national effort in Portugal to raise awareness of CKD and provide data-driven insights to support evidence-based public health policies.

Based on our findings, ≈5% of individuals in Portugal may have kidney disease, aligning with global CKD prevalence estimates [10]. The detection of albuminuria among individuals with no prior CKD diagnosis is particularly concerning, given that one-third of these individuals were <40 years of age. Younger individuals are frequently considered at low risk for CKD and therefore are less likely to be screened, leading to missed opportunities for early intervention. Without timely diagnosis, early-stage kidney damage may progress to advanced CKD, ultimately resulting in dialysis or kidney transplantation and contributing to the growing economic burden of CKD management. These findings underscore the importance of targeting younger populations through CKD awareness campaigns and screening initiatives.

In the awareness study, the prevalence of albuminuria was estimated at 3.4%. Although the response rate was 4.1%, slightly below the expected 5%, this component was primarily designed as an awareness-raising initiative rather than a diagnostic tool. Notably, our open, community-based approach contrasts with the structured methodology used in the Dutch THOMAS study [11], which achieved a participation rate of 44.3–59.4%, depending on the screening method used. While the THOMAS study demonstrated the feasibility of organized, mail-based screening, our findings highlight that decentralized, opportunistic distribution can still yield valuable epidemiological insights, especially when awareness is the primary goal.

It is important to recognize that large-scale screening efforts may increase the risk of false positives. This highlights the need for more sensitive and specific screening tools to ensure diagnostic accuracy and minimize the impact of false positive results, thereby optimizing healthcare resource allocation.

The use of a 0.12 g/l threshold for albuminuria based on urine test strip specifications offered a practical and standardized approach for large-scale screening. While this threshold may differ from clinical guidelines, it proved feasible for identifying potential cases in the population. Future research should compare these findings with laboratory-based analyses to validate their diagnostic accuracy and alignment with clinical thresholds.

The associations observed between albuminuria and established risk factors were consistent with the existing literature, reinforcing the need to prioritize high-risk groups for early and targeted screening. Importantly, lower education level was consistently associated with albuminuria across both studies and may serve as a surrogate marker for broader social and behavioural determinants of health, including sedentary lifestyle patterns, suboptimal dietary habits and reduced access to health-promoting resources.

Regional variation was also evident. Albuminuria prevalence was highest in the North. In those with unknown CKD, albuminuria was highest in the Algarve and Centre regions (screening study), and in Lisbon and Tagus Valley region (awareness study). These differences may reflect regional disparities in health-related behaviours, such as dietary patterns, or health practices. Differences in health access may justify the regional differences between known and unknown CKD. Addressing these differences could involve geographically tailored public health initiatives to mitigate risk and improve early detection.

This study had several limitations. First, the use of urine test strips, while suitable for large-scale screening, introduces inaccuracies and limitations in diagnostic precision due to their undefined sensitivity and specificity. This may have resulted in an overestimation of albuminuria prevalence. Further research is needed to validate these tools against laboratory-based diagnostics. Second, the door-to-door recruitment strategy may have introduced selection bias, excluding individuals who were not at home during data collection, potentially skewing the sample towards specific demographics. Likewise, the awareness study relied on convenience sampling, potentially overrepresenting individuals with pre-existing kidney health concerns. Lastly, the response rate in the awareness study was relatively low, with only 704 participants registering results out of the 17 000 test strips distributed. This limits the generalizability of our findings and underscores the need for improved community engagement strategies in future awareness efforts.

## CONCLUSIONS

This study highlights the importance of continued monitoring and targeted screening efforts, especially among high-risk populations, while also extending attention to individuals not traditionally considered at risk, as many remain undiagnosed with conditions such as diabetes or hypertension, and because CKD may also occur beyond these comorbidities. The findings emphasize that investments in CKD awareness and preventive public health strategies hold significant potential to mitigate the burden of CKD in Portugal.

The implementation of routine albuminuria screening in primary care settings for high-risk individuals appears essential. This approach enables earlier detection and timely intervention, with the potential to slow CKD progression and reduce associated cardiovascular complications, ultimately improving patient outcomes. Public awareness campaigns and integrating standardized, population-based screening protocols into existing healthcare infrastructures could represent a critical step toward mitigating the CKD epidemic in Portugal.

## Data Availability

The data that support the findings of this study are available upon reasonable request to the corresponding author. The data are not publicly available due to privacy or ethical restrictions.

## References

[1] Foreman KJ, Marquez N, Dolgert A et al. Forecasting life expectancy, years of life lost, and all-cause and cause-specific mortality for 250 causes of death: reference and alternative scenarios for 2016–40 for 195 countries and territories. Lancet 2018;392:2052–90. 10.1016/S0140-6736(18)31694-5 30340847 PMC6227505

[2] Boerstra BA, Boenink R, Astley ME et al. The ERA Registry annual report 2021: a summary. Clin Kidney J 2024;17:sfad281. 10.1093/ckj/sfad281 38638342 PMC11024806

[3] Sociedade Portuguesa Nefrologia . Registo Português Da Doença Renal Cronica Sob Tratamento De Substituição Renal. 2023. https://www.spnefro.pt/assets/relatorios/tratamento_doenca_terminal/registo-nacional-de-doenca-renal-cronicav4-apresentacao-dmr.pdf [accessed].

[4] Bakris GL, Agarwal R, Anker SD et al. Effect of finerenone on chronic kidney disease outcomes in type 2 diabetes. N Engl J Med 2020;383:2219–29. 10.1056/NEJMoa2025845 33264825

[5] Heerspink HJL, Stefánsson BV, Correa-Rotter R et al. Dapagliflozin in patients with chronic kidney disease. N Engl J Med 2020;383:1436–46. 10.1056/NEJMoa2024816 32970396

[6] Herrington WG, Staplin N, Wanner C et al. Empagliflozin in patients with chronic kidney disease. N Engl J Med 2023;388:117–27. 10.1056/NEJMoa2204233 36331190 PMC7614055

[7] Kidney Disease: Improving Global Outcomes CKD Work Group . KDIGO 2024 clinical practice guideline for the evaluation and management of chronic kidney disease. Kidney Int 2024;105(4 Suppl):S117–314. 10.1016/j.kint.2023.10.018 38490803

[8] Pouwels X, van Mil D, Kieneker LM et al. Cost-effectiveness of home-based screening of the general population for albuminuria to prevent progression of cardiovascular and kidney disease. EClinicalMedicine 2024;68:102414. 10.1016/j.eclinm.2023.102414 38299045 PMC10827681

[9] Lamprea-Montealegre JA, Estrella MM. Population-wide albuminuria screening: implications for CKD detection and management. Lancet 2023;402:1020–1. 10.1016/S0140-6736(23)01140-6 37597525

[10] Jager KJ, Kovesdy C, Langham R et al. A single number for advocacy and communication-worldwide more than 850 million individuals have kidney diseases. Kidney Int 2019;96:1048–50. 10.1016/j.kint.2019.07.012 31582227

[11] van Mil D, Kieneker LM, Evers-Roeten B et al. Participation rate and yield of two home-based screening methods to detect increased albuminuria in the general population in the Netherlands (THOMAS): a prospective, randomised, open-label implementation study. Lancet 2023;402:1052–64. 10.1016/S0140-6736(23)00876-0 37597522

